# Preconception lifestyle intervention reduces long term energy intake in women with obesity and infertility: a randomised controlled trial

**DOI:** 10.1186/s12966-018-0761-6

**Published:** 2019-01-08

**Authors:** T. M. van Elten, M. D. A. Karsten, A. Geelen, R. J. B. J. Gemke, H. Groen, A. Hoek, M. N. M. van Poppel, T. J. Roseboom

**Affiliations:** 1 Department of Public and Occupational Health, Amsterdam UMC, Vrije Universiteit Amsterdam, VU University medical centre, de Boelelaan 1117, Amsterdam, The Netherlands; 2 Department of Clinical Epidemiology, Biostatistics and Bioinformatics, Amsterdam UMC, University of Amsterdam, Academic Medical Centre, Meibergdreef 9, Amsterdam, The Netherlands; 3 Department of Obstetrics and Gynaecology, Amsterdam UMC, University of Amsterdam, Academic Medical Centre, Meibergdreef 9, Amsterdam, The Netherlands; 4 Amsterdam Public Health Research Institute, Amsterdam, The Netherlands; 5 Amsterdam Reproduction and Development, Amsterdam, The Netherlands; 6 Department of Obstetrics and Gynaecology, University of Groningen, University Medical Centre Groningen, Groningen, the Netherlands; 7 Division of Human Nutrition, Wageningen University & Research, Wageningen, The Netherlands; 8 Department of Paediatrics, Amsterdam UMC, Vrije Universiteit Amsterdam, VU University medical centre, de Boelelaan 1117, Amsterdam, The Netherlands; 9 Department of Epidemiology, University of Groningen, University Medical Centre Groningen, Groningen, the Netherlands; 10 University of Graz, Institute of Sport Science, Graz, Austria

**Keywords:** Lifestyle intervention program, Diet, Diet quality, Physical activity, Accelerometers, Obesity, Weight loss, Long term follow-up

## Abstract

**Background:**

The preconceptional period may be an optimal window of opportunity to improve lifestyle. We previously showed that a 6 month preconception lifestyle intervention among women with obesity and infertility was successful in decreasing the intake of high caloric snacks and beverages, increasing physical activity and in reducing weight in the short term. We now report the effects of the preconception lifestyle intervention on diet, physical activity and body mass index (BMI) at 5.5 years (range = 3.7–7.0 years) after the intervention.

**Methods:**

We followed women who participated in the LIFEstyle study, a multicentre RCT in which women with obesity and infertility were assigned to a six-month lifestyle intervention program or prompt infertility treatment (*N* = 577). Diet and physical activity 5.5 years later were assessed with an 173-item food frequency questionnaire (*N* = 175) and Actigraph triaxial accelerometers (*N* = 155), respectively. BMI was calculated from self-reported weight and previously measured height (*N* = 179). Dietary intake, physical activity, and BMI in the intervention and control group were compared using multivariate regression models. Additionally, dietary intake, physical activity and BMI of women allocated to the intervention arm with successful weight loss during the intervention (i.e. BMI < 29 kg/m^2^ or ≥ 5% weight loss), unsuccessful weight loss and the control group were compared with ANCOVA.

**Results:**

Although BMI did not differ between the intervention and control group 5.5 years after the intervention (− 0.5 kg/m^2^ [− 2.0;1.1]; *P* = 0.56), the intervention group did report a lower energy intake (− 216 kcal/day [− 417;-16]; *P* = 0.04). Women in the intervention arm who successfully lost weight during the intervention had a significantly lower BMI at follow-up compared to women in the intervention arm who did not lose weight successfully (− 3.4 kg/m^2^ [− 6.3;-0.6]; *P* = 0.01), and they reported a significantly lower energy intake compared to the control group (− 301 kcal [− 589;-14]; *P* = 0.04). Macronutrient intake, diet quality, and physical activity did not differ between the intervention and control group, irrespective of successful weight loss during the intervention.

**Conclusions:**

In our study population, a preconception lifestyle intervention led to reduced energy intake 5.5 years later. Additionally, women allocated to the intervention group who were successful in losing weight during the intervention also had a lower BMI at follow-up. This shows the potential sustainable effect of a preconception lifestyle intervention.

**Trial registration:**

This trial was registered on 16 November 2008 in the Dutch trial register; clinical trial registry number NTR1530.

**Electronic supplementary material:**

The online version of this article (10.1186/s12966-018-0761-6) contains supplementary material, which is available to authorized users.

## Background

Obesity is one of the greatest public health problems [1, 2]. The prevalence of obesity has tripled in many European countries since the 1980’s [1, 3], ranging from 10 to 30% in the adult population [4]. Obesity is a major risk factor for developing non-communicable diseases, including cardiovascular diseases, diabetes and cancer [5–7]. Furthermore, obesity is adversely associated with women’s reproductive health [8].

Guidelines recommend lifestyle modification as the first step in the management of obesity [9]. However, changing lifestyle is difficult and most lifestyle interventions, if effective, result in only modest short term changes [10–12]. Evidence regarding long term lifestyle change following interventions is scarce: Only few studies have reported long term dietary and physical activity changes besides long term weight changes [13–15].

Although changing lifestyle and maintaining those changes is difficult, the period before and during pregnancy may be an optimal period to intervene. Reproduction is a life period which motivates women to adopt health optimizing behaviours, with the perspective of the health and well-being of their unborn child [16]. Lifestyle changes, for example to stop smoking and/or drinking alcohol, are more successful among pregnant women or women with a wish to conceive [17–19].

We previously showed that a six-month preconception lifestyle intervention program reduced the intake of high caloric snacks and beverages and increased physical activity among women with obesity and infertility [20]. These relatively small improvements in diet led to important improvements in cardiometabolic health by halving the odds of metabolic syndrome [21].

Maintaining a healthy lifestyle in the long term is notoriously difficult. We therefore investigated the effects of the preconception lifestyle intervention on diet, physical activity and body mass index at (BMI) 5.5 years (range = 3.7–7.0 years) after the intervention. We hypothesised that a preconception lifestyle intervention led to prolonged healthier dietary intake, more physical activity and a lower BMI. Additionally, we hypothesised that the effect of the intervention on lifestyle is more pronounced among women allocated to the intervention group who were successful in losing weight during the intervention.

## Methods

This paper comprises the follow-up of a multicentre randomised controlled trial (RCT). Between June 2009 and June 2012, 577 women with obesity and infertility aged 18 to 39 years were allocated to the intervention or control group. Women in the intervention group received a six-month structured lifestyle program. When the target weight reduction was met or when BMI decreased below 29 kg/m^2^ or after finalisation of the six-month program, infertility treatment was started [22]. The control group received immediate infertility treatment as usual. The design and main results of the LIFEstyle RCT have been published previously [22, 23]. In brief, the lifestyle intervention did not result in higher rates of vaginal birth of a healthy singleton at term in the intervention group within 24 months after randomisation. Women in the intervention group had significantly more ongoing pregnancies that resulted from natural conceptions [23]. Three to 8 years after inclusion in the LIFEstyle RCT, all women were approached to participate in the follow-up study, designated as the WOMB project [24]. The follow-up assessments included questionnaires about current lifestyle and health. Furthermore, accelerometers were worn to assess physical activity. More details about this follow-up study have been published elsewhere [24].

The LIFEstyle study as well as the WOMB project were conducted according to the guidelines laid down in the Declaration of Helsinki and all procedures were approved by the Medical Ethics Committee of the University Medical Centre Groningen, the Netherlands (METc 2008/284). Written informed consent was obtained from all participants at the start of the LIFEstyle study and at the start of the WOMB project.

### Intervention

The six-month structured lifestyle intervention program aimed at a weight loss of at least 5% of the original body weight or a BMI below 29 kg/m^2^. The program consisted of dietary counselling, encouragement to increase physical activity and an individualised behavioural modification plan [25]. Six face-to-face consultations and four telephone or e-mail consultations with trained intervention coaches were scheduled. Women were advised a healthy diet with a caloric restriction of 600 kcal/day compared to their habitual intake, but not below 1200 kcal/day. They received feedback on their diet using a web-based food diary of the Netherlands Nutrition Centre [26]. This food diary was used for counselling purposes and mean caloric intake per day was recorded during the intervention on each consultation. In addition, women were advised to be moderately physically active for at least two to three times a week with a minimum of 30 min/day, and to increase their physical activity by taking at least 10,000 steps/day. Women were instructed to monitor their step count using a daily worn pedometer. A physical activity diary was kept to establish self-monitoring.

### Control condition

Women allocated to the control group started with prompt infertility treatment and were treated according to the Dutch infertility guidelines [27], irrespective of their BMI. They did not receive lifestyle counselling. Both groups received a patient information leaflet as part of the informed consent procedure regarding the negative effects of overweight and obesity on their reproductive health, pregnancy, and pregnancy outcomes.

### Diet

Dietary intake 5.5 years after randomisation was examined using a validated semi-quantitative 173-item Food Frequency Questionnaire (FFQ) [28], asking about frequency and consumed amounts over a 1 month reference period. In this study we report total energy intake, total fat, saturated fat, protein, carbohydrate and fibre intake using the Dutch Food Composition Database of 2016 [29]. Furthermore, the Dutch Healthy Diet index 2015 (DHD15-index) was calculated using the 173-item FFQ. The DHD15-index score and its 15 components were based on the guidelines as described by Looman et al. [30]. The DHD15-index is a score reflecting the adherence to the 2015 Dutch guidelines for a healthy diet [31]. For each separate component scoring ranged from 0 to 10, resulting in a total score between 0 (no adherence) to 150 (complete adherence). The DHD15-index in this paper includes 13 of the 15 components: vegetables, fruit, wholegrain products, legumes, nuts, dairy, fish, tea, fats and oils, red meat, processed meat, sugary sweetened beverages and fruit juices, and alcohol. We were not able to calculate the sodium component, as self-report methods like FFQs are not able to estimate salt intake sufficiently. Furthermore, we could not calculate the coffee component since the FFQ we used did not distinguish between filtered and unfiltered coffee. This resulted in a total score ranging from 0 (no adherence) to 130 (complete adherence).

### Physical activity

Physical activity was measured with the triaxial Actigraph wGT3X-BT or GT3X+ [32]. Women were asked to wear an accelerometer for seven consecutive days on their right hip by an elastic waist belt, from the moment they got out of bed until the moment they went to bed. Sampling frequency of the accelerometers was set at 80 hertz and epochs of 10 s [33, 34]. Women were instructed to take the accelerometer off during bathing, showering or swimming activities. In addition, women were asked to write down why and when the accelerometer was taken off in a daily activity log. Every morning they received a text message by telephone to increase compliance.

### Body mass index

Current weight of women was asked using a questionnaire. Height was measured during the intervention by trained research nurses that were not involved in the lifestyle intervention coaching. BMI was calculated by dividing weight in kilograms by the square of the height in meters.

### Statistical analysis

Baseline characteristics were displayed as means and standard deviations (SD) or as medians and interquartile ranges (IQR) for continuous variables, and as percentage and number of participants (N) for categorical data. Independent sample Student’s t-tests, Mann-Whitney U-tests and Chi-square tests were used to compare both groups as appropriate. We additionally compared the participants within the follow-up with the non-participants, using the same statistical methods, to check for selective participation in the follow-up.

The Goldberg cut-off [35] was used to check for over- and underreporting of energy intake at individual level using the Schofield formula to calculate basal metabolic rate (BMR). When energy intake divided by BMR was < 0.87 or > 2.75 these values were considered as outliers. In our data, we observed underreporting of energy intake (in 25.7% of the women), which is in line with other studies in obese people [36, 37]. We performed a sensitivity analysis excluding all underreporters.

For the accelerometers, crude data was obtained using ActiLife 6 (ActiGraph, LLC, Pensacola, Florida, USA). During data cleaning, the default settings of Choi 2011 were used [38], defining a non-wear period as no counts for at least 90 min. Women with at least 3 valid days, including at least 10 h of wear time per day, were included in the analysis (% of women with 3 valid wear days: 2.6%; 4 valid wear days: 9.7%; 5 valid wear days: 10.3%; 6 valid wear days: 21.9%; 7 valid wear days: 55.5%) [33, 34]. In addition, wear periods defined by ActiLife were compared with wear periods according to the participant’s activity log. Wear periods were manually adjusted when ActiLife incorrectly defined time as (non-)wear period (e.g. short periods of movement registration when the participant already stopped wearing the accelerometer). Freedson cut-off points [39] were used to determine the number of minutes per day of light (100–1951 cpm), moderate (1952–5724 cpm) and vigorous physical activity (> 5724 cpm). For the analysis, we included the time spent in total physical activity (PA) as percentage of total wear time, and total moderate to vigorous physical activity (MVPA) in minutes per day. We additionally performed a sensitivity analysis including only women with at least 3 valid days, of which at least 1 valid weekend day.

Differences in dietary intake, physical activity and BMI between the intervention and the control group 5.5 years after randomisation were analysed by multivariate linear regression models, adjusted for the following covariates: Caucasian origin (yes/no), education level (categorical: no education or primary school; secondary education; intermediate vocational education; higher vocational education and university), smoking (yes/no) and duration of infertility (months). The results are reported as differences and corresponding 95% confidence intervals (C.I.). To study if the intervention effect on lifestyle is more pronounced among women allocated to the intervention arm who were successful in losing weight during the intervention (BMI < 29 kg/m^2^ or ≥ 5% weight reduction), we determined whether dietary intake, physical activity and BMI differed between women allocated to the intervention group who successfully lost weight during the 6 month intervention, women allocated to the intervention group who did not successfully lose weight, and women allocated to the control group using ANCOVA. If between group differences were present, Tukey post-hoc test was used to test the within group differences. We corrected for the same covariates as mentioned previously. Additionally, we performed a sensitivity analysis excluding women in the control group who lost weight successfully during the first 6 months after randomisation (*N* = 3) and excluding women in the control group with missing data on weight loss (*N* = 16 for energy intake; *N* = 22 for BMI at follow-up).

In a subgroup of women (*N* = 101) height and weight were measured by researchers. To compare the self-reported BMI used for the current study with measured BMI, we calculated the Pearsons correlation coefficient.

Statistical analyses were performed using the software Statistical Package for the Social Sciences (SPSS) version 24 for Windows (SPSS, Chicago, IL, USA). *P*-values < 0.05 were considered statistically significant.

## Results

**Fig. 1:**
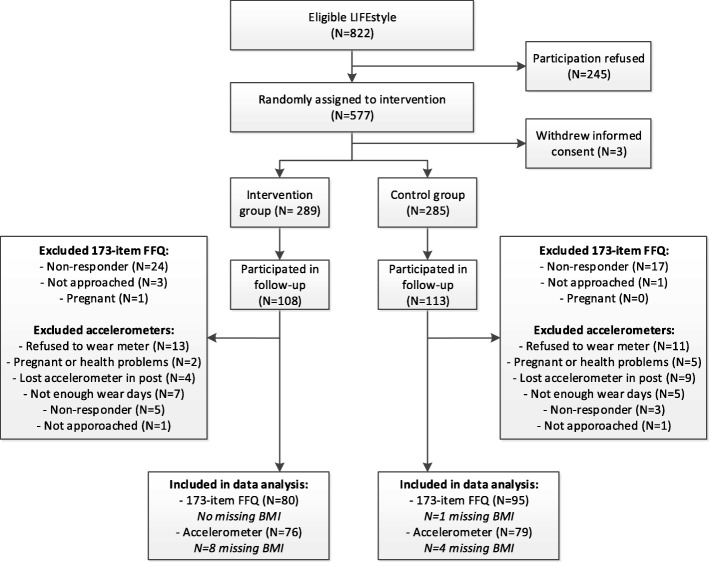
Flowchart of participants, BMI is self-reported and was missing in case of missing current weight. In total, 64 women in the intervention group and 74 women in the control group had data on both the FFQ and the accelerometers; 16 women in the intervention group and 21 women in the control group had FFQ data but no accelerometer data; 12 women in the intervention group and 5 women in the control group had accelerometer data but no FFQ data

In total 221 women, of the 577 women randomised in the trial (38.3%), participated in the follow-up. In the follow-up study, 180 women (81.4%) wore an accelerometer, of these women we were able to include a total of 155 women (86.1% of 180 women) in our analyses. The FFQ was filled out by 175 women (79.2% of 221 women) and all women were included in our analyses (Fig. 1). Mean duration of follow-up in the total study population was 5.5 years, with a minimum of 3.7 years and a maximum of 7.0 years. Compared to the original study population, women participating in the follow-up were older at time of randomisation, more often of Caucasian origin, had a shorter duration of infertility and were more often successful in losing weight during the intervention (Additional file 1). Furthermore, women who did not wear the accelerometer had on average a 2 months shorter time since completion of the first 6 months of the LIFEstyle study, compared to women who did wear the accelerometer. In the current follow-up study, women who were randomised into the intervention group (*N* = 92) did not differ in baseline characteristics compared to the women in the control group (*N* = 100), with exception of duration of infertility (22 months in the intervention group vs. 17 months in the control group; *P* = 0.02; Table 1).

**Table 1:** Baseline characteristics of women who filled out the 173-item FFQ and/or wore an accelerometer^a^

	Intervention (*N* = 92)	Control (*N* = 100)	*P*-value
Age (mean; SD)	30.4 (4.1)	29.9 (4.5)	0.43
Caucasian (%; N)	93.5 (86)	95.0 (95)	0.65
Body Mass Index (kg/m^2^; mean; SD)^b^	35.9 (3.3)	35.8 (3.1)	0.73
Education level (%; N)			
No education or primary school (4–12 years)	4.5 (4)	1.0 (1)	0.52
Secondary education	20.2 (18)	21.4 (21)	
Intermediate Vocational Education	51.7 (46)	55.1 (54)	
Higher Vocational Education and University	23.6 (21)	22.4 (22)	
Smoking (yes; %; N)	22.0 (20)	17.2 (17)	0.40
Nulliparous (yes; %; N)	75.0 (69)	73.0 (73)	0.75
Anovulatory (yes; %; N)	46.7 (43)	53.0 (53)	0.39
PCOS (yes; %; N)	35.9 (33)	42.0 (42)	0.38
Duration infertility (months; median; IQR)	22.0 (15.0; 37.0)	17.0 (13.0; 25.8)	0.02
Pregnant after randomisation (yes; %; N)^c^	68.5 (63)	76.0 (76)	0.24

^a^For continuous data independent sample Student’s t-tests and for categorical data Chi-square tests were used to compare both groups. PCOS = Polycystic Ovary Syndrome

^b^BMI was measured by research nurses during hospital visit

^c^Pregnancy of at least 24 weeks

At follow-up, the intervention group reported a statistically significantly lower energy intake compared to the control group (− 216 kcal [95%C.I. − 417; − 16]; *P* = 0.04; Table 2), while there were no differences in macronutrient intake as percentage of total energy, diet quality measured with the DHD15-index, and physical activity. Excluding underreporters did not affect the results for energy intake. Despite the difference in reported energy intake at follow-up, we did not observe a difference in BMI 5.5 years after randomisation between the intervention and the control group (− 0.5 kg/m^2^ [− 2.0; 1.1]; *P* = 0.56). In line with the lower reported energy intake, the intervention group had a significantly lower absolute intake of all macronutrients at follow-up, with exception of protein (− 6.7 g [95%C.I. -13.7; 0.4]; *P* = 0.06) and saturated fat (− 3.3 g [95%C.I. -7.2; 0.5]; *P* = 0.09).

**Table 2:** Differences in BMI, dietary intake, diet quality and physical activity between intervention and control group^a^

	N	Intervention	N	Control	β (95% CI) unadjusted	*P*-value	β (95% CI) adjusted^c^	*P*-value
BMI (mean; SD)^b^	84	34.4 (5.1)	95	34.5 (5.0)	−0.1 (−1.6; 1.4)	0.86	−0.5 (−2.0; 1.1)	0.56
Energy (kcal; mean; SD)	80	1749 (561)	95	1973 (690)	− 224 (− 414; −34)	0.02	− 216 (−417; − 16)	0.04
Energy without underreporters (kcal; mean; SD)	57	1992 (453)	73	2222 (556)	− 231 (− 410; −51)	0.01	− 200 (− 389; − 11)	0.04
Protein (en%; mean; SD)	80	16.2 (3.1)	95	15.9 (2.9)	0.3 (−0.6; 1.1)	0.56	0.3 (−0.6; 1.2)	0.55
Carbohydrates (en%; mean; SD)	80	44.3 (7.0)	95	44.3 (7.0)	−0.01 (−2.1; 2.1)	> 0.99	0.4 (−1.8; 2.5)	0.74
Fat (en%; mean; SD)	80	35.6 (6.3)	95	36.4 (6.4)	−0.8 (−2.7; 1.1)	0.40	−1.1 (−3.0; 0.9)	0.27
Saturated fat (en%; mean; SD)	80	12.7 (2.6)	95	12.8 (3.2)	−0.2 (−1.0; 0.7)	0.72	−0.2 (−1.1; 0.8)	0.73
Fibre (gram/MJ; mean; SD)	80	2.6 (0.7)	95	2.6 (0.6)	0.04 (−0.2; 0.2)	0.72	0.01 (−0.2; 0.2)	0.94
DHD15-index score	80	70.7 (14.6)	95	71.6 (14.1)	−0.9 (−5.2; 3.4)	0.67	−1.4 (−5.8; 3.0)	0.54
Total PA (% wear time; mean; SD)	76	27.6 (6.6)	79	27.4 (6.7)	0.2 (−1.9; 2.3)	0.86	−0.4 (−2.5; 1.7)	0.71
MVPA (min/day; mean; SD)	76	32.0 (15.4)	79	33.2 (18.4)	−1.2 (−6.6; 4.2)	0.67	−1.0 (−6.6; 4.5)	0.72
	N	Intervention	N	Control	OR (95% CI) unadjusted	*P*-value	OR (95% CI) adjusted^c^	*P*-value
Meeting the Dutch PA guidelines (yes; %; N)	76	65.8 (50)	79	63.3 (50)	1.1 (0.6; 2.2)	0.75	1.2 (0.6; 2.3)	0.66

^a^Differences in BMI, dietary intake, diet quality and physical activity 5.5 years after randomization between the intervention and the control group were analysed by linear regression models, with the exception of meeting the Dutch physical activity guidelines which is analysed by logistic regression. SD = standard deviation; kcal = kilocalories; en% = percentage of total energy intake; DHD 15-index score = Dutch Healthy Diet index 2015; PA = physical activity; MVPA = moderate to vigorous physical activity; min/day = minutes per day

^b^BMI is self-reported

^c^Adjusted for: Caucasian origin (yes/no), education level (categorical: no education or primary school; secondary education; intermediate vocational education; higher vocational education and university), smoking (yes/no) and duration of infertility (months)

**Fig. 2:**
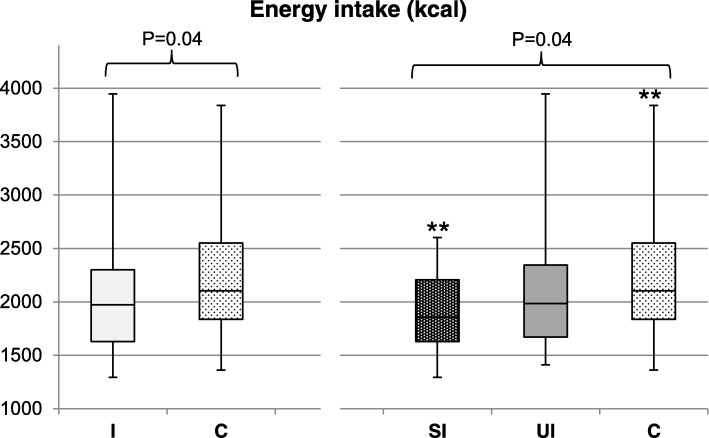
Differences in energy intake (kcal) without underreporters at follow-up. Differences between women allocated to the intervention group (I; *N* = 73) versus the control group (C; *N* = 57) were analysed using multivariate linear regression, corrected for: Caucasian origin (yes/no), education level (categorical: no education or primary school; secondary education; intermediate vocational education; higher vocational education and university), smoking (yes/no) and duration of infertility (months). Differences among women who successfully lost weight during the intervention (SI; *N* = 29), who were unsuccessful in losing weight (UI; *N* = 24) and the control group (C; *N* = 73). were analysed using ANCOVA, corrected for the previously mentioned covariates. Tukey post-hoc tests were used to analyse differences within groups. Mean kcal and SD: *I* = 1992 kcal (453); C = 2222 kcal (556); SI = 1917 kcal (358); UI = 2097 kcal (544); C = 2222 kcal (556). **P-value = 0.04

**Fig. 3:**
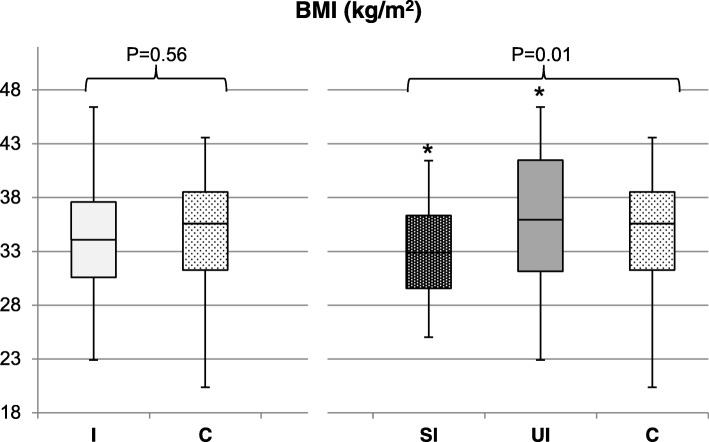
Differences in self-reported BMI (kg/m^2^) at follow-up. Differences between women allocated to the intervention group (I; *N* = 84) versus the control group (C; *N* = 95) were analysed using multivariate linear regression, corrected for: Caucasian origin (yes/no), education level (categorical: no education or primary school; secondary education; intermediate vocational education; higher vocational education and university), smoking (yes/no) and duration of infertility (months). Differences among women who successfully lost weight during the intervention (SI; *N* = 45), who were unsuccessful in losing weight (UI; *N* = 33) and the control group (C; *N* = 95) were analysed using ANCOVA, corrected for the previously mentioned covariates. Tukey post-hoc tests were used to analyse differences within groups. Mean BMI and SD: *I* = 34.4 kg/m^2^ (5.1); C = 34.5 kg/m^2^ (5.0); SI = 32.9 kg/m^2^ (4.0); UI = 36.2 kg/m^2^ (6.0); C = 34.5 kg/m^2^ (5.0). *P-value = 0.01

Reported energy intake and BMI at follow up differed significantly among women in the intervention group who successfully lost weight during the intervention, women in the intervention group who did not lose weight successfully, and the control group (*P* = 0.04 and *P* = 0.01, respectively; Fig. 2 and Fig. 3). Post-hoc analysis showed no difference in reported energy intake between women allocated to the intervention group who successfully lost weight during the intervention compared to women allocated to the intervention group who did not lose weight successfully (1917 kcal [SD: 358] versus 2097 kcal [SD: 545]; *P* = 0.44; Fig. 2). However, they reported a significantly lower energy intake compared to the control group (1917 kcal [SD: 358] versus 2222 kcal [SD: 556]; *P* = 0.04). BMI in women who successfully lost weight during the intervention was significantly lower compared to women who did not lose weight successfully (32.9 kg/m^2^ [SD: 4.0] versus 36.2 kg/m^2^ [SD: 6.0]; *P* = 0.01; Fig. 3), and compared to the control group, although this difference was not statistically significant (32.9 kg/m^2^ [SD: 4.0] versus 34.5 kg/m^2^ [SD: 5.0]; *P* = 0.13). No differences between the three groups were observed for macronutrient intake, diet quality and physical activity (results not shown). Results of our sensitivity analysis excluding women in the control group who lost weight successfully during the first 6 months after randomisation (*N* = 3) and excluding women in the control group with missing data on weight loss (*N* = 16 for energy intake; *N* = 22 for BMI at follow-up) did not change the conclusion regarding BMI. However, the difference in energy intake was no longer statistically significant after adjusting for covariates (*P* = 0.08; Additional file 2: Figure S1 and Additional file 3: Figure S2).

Measured and self-reported BMI were highly correlated (0.90 in women allocated to the intervention group who successfully lost weight, 0.88 in women allocated to the intervention group who did not lose weight successfully and 0.92 in the control group). ANOVA analysis of differences in BMI between the groups of women allocated to the intervention group who were or were (not) successful in losing weight during the intervention and the control group were similar to the results with measured BMI compared to self-reported BMI (results not shown).

Sensitivity analysis regarding differences in physical activity, including only women with at least 3 valid days of accelerometer data of which at least 1 valid weekend day (*N* = 137) showed similar results compared to the total study population (results not shown).

## Discussion

In addition to our earlier finding that a 6 month preconception lifestyle intervention successfully improved lifestyle in the short term, we now show it also reduced energy intake 5.5 years later in our study population. Furthermore, women allocated to the intervention arm who were successful in losing weight during the intervention had a lower BMI and reported a lower energy intake compared to women allocated to the intervention arm who were not successful in losing weight and compared to women in the control group. This indicates that women allocated to the intervention arm who were successful in short term weight loss are more likely to successfully change their lifestyle and BMI in the long term.

Although the intervention successfully lowered reported energy intake in the long term in the intervention group compared to the control group, this was not reflected in BMI. We do not know why the reduction in reported energy intake is not reflected in BMI. Underreporting of energy intake might play a role [36, 37]. However, adjustment for underreporting using the Goldberg cut off did not rule out the intervention effect on energy intake. Women who successfully lost weight during the intervention reported a reduced energy intake and had a lower BMI, suggesting that among these women the intervention had long lasting beneficial effects on lifestyle that led to significantly reduced BMI. Additionally, we explored whether women allocated to the intervention arm who were successful in reducing their energy intake during the intervention (≥600 kcal compared to their baseline intake) had a lower energy intake and BMI at follow-up. This was not the case, which implies that a lower reported energy intake and BMI at follow-up were predicted by successful weight loss during the intervention and not by a reported reduction in energy intake during the intervention.

Among women allocated to the intervention arm who did not lose weight successfully during the intervention, the intervention did not affect diet, physical activity and BMI in the long term. Therefore, underlying reasons for unsuccessful lifestyle change should be investigated more thoroughly in future research. For example, besides a randomised comparison between groups, regression models can be used to examine which determinants are associated with successful lifestyle change [40]. This may help design more effective interventions that will help women to achieve a sustainable healthy lifestyle and weight.

Although the intervention initially appeared to have a positive effect on physical activity there was no effect on physical activity in the long term. Accelerometers only capture highly dynamic activities. Cycling activities are not well measured by accelerometers and we instructed women to take off the accelerometer while swimming [41]. In our data, we however did not observe any difference in self-reported cycling or swimming activities between the intervention and control group. Measurement error could still be present in our data, although we do not expect this to be different between the intervention and control group. Little is known about maintaining changes in physical activity over longer periods of time [42, 43]. We speculate that adding physical activity into daily routine takes more effort compared to incorporating lasting changes in habitual diet, especially during pregnancy or when having young children [44]. The lack of intervention effect on physical activity might also explain the lack of intervention effect on BMI. If the intervention would have increased physical activity in the long term, it might have also led to reduced BMI.

This is the first paper describing long term effects of a preconception lifestyle intervention on dietary intake and physical activity among women with obesity in an experimental setting. The period around pregnancy can be seen as a teachable moment, which can motivate women to change their lifestyle [16]. Literature regarding the long term effects of preconception lifestyle interventions on dietary intake and physical activity is scarce and inconsistent. The few preconception lifestyle interventions that have been performed only include short term follow-up [45–47]. One preconception trial with a follow-up of 12 months showed that the intervention group had a significant lower body weight and BMI compared to the control group [48]. However, this study did not examine dietary intake and only assessed whether the participants met the recommended physical activity guidelines. Our results are promising, since we showed that a preconception lifestyle intervention reduced reported energy intake in the long term. Hence, if women with obesity successfully lose weight preconceptionally the beneficial effects on lowering energy intake and BMI appear to have prolonged effects, suggesting a potentially sustainable effect of preconception lifestyle interventions. These changes in energy intake and BMI may not only improve women’s health but their offspring’s health too [49, 50].

One of our limitations, and in general for nutritional research, is the use of questionnaires to report dietary intake. People with obesity tend to underreport their total energy intake [36, 37]. However, it is unlikely that the observed effect can only be explained by underreporting, since: 1) excluding women who underreported their energy intake did not change the associations, 2) the successful women allocated to the intervention arm, who reported a significantly lower energy intake, also had a lower BMI. Furthermore, we cannot exclude the possibility that social desirability bias explains the observed intervention effect on reported energy intake. A second limitation is the use of self-reported BMI in our study. Women with obesity generally underreport their BMI [51]. However, self-reported and measured BMI in our data were highly correlated.

Our follow-up study has a low response rate (38.3%) [52], which led to selective participation. This selective participation might have influenced our results. Women who were successful in losing weight during the intervention were more likely to participate in our follow-up study (Additional file 1). Therefore, our results may not be generalisable to our entire study population. Furthermore, women who did not wear an accelerometer had on average a 2 months shorter time since completion of the LIFEstyle intervention period compared to women who did participate (Additional file 1). Although statistically significant, it is unlikely that this 2 months difference in time between completion of the intervention affected the generalisability of our results. We found no evidence that selective participation of older women, women of Caucasian origin and women with a shorter duration of infertility biased our results, since adjustment for these characteristics that differed between participants and non-participants (Additional file 1) did not influence our findings. We studied a group of women with infertility, and therefore our findings initially apply only to women with infertility. Further studies should investigate whether preconception lifestyle interventions are equally successful in women who are fertile.

To conclude, a preconception lifestyle intervention led to reduced energy intake at 5.5 years after the intervention in our study population. Additionally, women allocated to the intervention group who successfully lost weight during the intervention reported a lower energy intake and a reduced BMI in the long term compared to women allocated to the intervention group who did not successfully lose weight and to women in the control group. These results show the potential sustainable effect of a preconception lifestyle intervention.

## Additional files

Additional file 1: Differences between baseline characteristics of participants versus the non-participants. (PDF 164 kb)

Additional file 2: **Figure S1.** Differences in energy intake (kcal) at follow-up without underreporters and without women who successfully lost weight in the control group (*N* = 3) or had missing data on weight loss in the control group (*N* = 16). Differences among women who successfully lost weight during the intervention (SI; *N* = 29), who were unsuccessful in losing weight during the intervention (UI; *N* = 24) and women who were unsuccessful in losing weight in the control group (UC; *N* = 54) were analysed using ANCOVA, corrected for: Caucasian origin (yes/no), education level (categorical: no education or primary school; secondary education; intermediate vocational education; higher vocational education and university), smoking (yes/no) and duration of infertility (months). Tukey post-hoc tests were used to analyse differences within groups. Mean kcal and SD: SI = 1917 kcal (358); UI = 2097 kcal (544); UC = 2234 kcal (583). (PDF 7 kb)

Additional file 3: **Figure S2.** Differences in self-reported BMI (kg/m^2^) at follow-up without women who successfully lost weight in the control group (N = 3) or had missing data on weight loss in the control group (*N* = 22). Differences among women who successfully lost weight during the intervention (SI; *N* = 45), who were unsuccessful in losing weight during the intervention (UI; *N* = 33) and women who were unsuccessful in losing weight in the control group (UC; *N* = 70) were analysed using ANCOVA, corrected for: Caucasian origin (yes/no), education level (categorical: no education or primary school; secondary education; intermediate vocational education; higher vocational education and university), smoking (yes/no) and duration of infertility (months). Tukey post-hoc tests were used to analyse differences within groups. Mean BMI and SD: SI = 32.9 kg/m^2^ (4.0); UI = 36.2 kg/m^2^ (6.0); UC = 34.0 kg/m^2^ (4.8). * *P*-value = 0.01 (PDF 7 kb)

## Abbreviations

ANOVA: Analysis of Variance
BMI: Body Mass Index
CI: Confidence Interval
DHD15-index: Dutch Healthy Diet 2015 index
FFQ: Food Frequency Questionnaire
IQR: Inter Quartile Range
MVPA: Moderate to Vigorous Physical Activity
PA: Physical Activity
PCOS: Polycystic Ovary Syndrome
RCT: Randomised Controlled Trial
SD: Standard Deviation
