# Supplementary material for: Preconception lifestyle intervention reduces long term energy intake in women with obesity and infertility: a randomised controlled trial
Source: Int J Behav Nutr Phys Act. 2019 Jan 8;16:3. doi: 10.1186/s12966-018-0761-6 (PMC6325811; doi:10.1186/s12966-018-0761-6)
Supplement: Supplementary file 1 — Differences between baseline characteristics of participants versus the non-participants. (PDF 164 kb) [file 12966_2018_761_MOESM1_ESM.pdf]

Additional file 1. Differences between baseline characteristics of participants versus the non-participants.*

	Non-participants 173-item FFQ (N=399)	Participants 173-item FFQ (N=175)	P-value	Non-participants accelerometer (N=419)	Participants accelerometer (N=155)	P-value
Age (mean; SD)	29.7 (4.7)	30.0 (4.2)	0.46	29.5 (4.7)	30.3 (4.2)	0.048
Caucasian (%; N)	84.0 (335)	95.4 (167)	<0.001	85.2 (357)	93.5 (145)	0.007
Body Mass Index (kg/m^2; mean; SD)**	36.1 (3.5)	36.0 (3.2)	0.74	36.1 (3.5)	35.9 (3.2)	0.43
Education level (%; N)						
No education or primary school (4-12 years)	5.8 (22)	2.9 (5)	0.11	5.5 (22)	3.3 (5)	0.54
Secondary education	25.9 (98)	19.4 (33)		24.8 (99)	21.3 (32)	
Intermediate Vocational Education	45.6 (173)	54.7 (93)		47.1 (188)	52.0 (78)	
Higher Vocational Education and University	22.7 (86)	22.9 (39)		22.6 (90)	23.3 (35)	
Smoking (yes; %; N)	25.5 (101)	20.2 (35)	0.18	24.8 (103)	21.6 (33)	0.43
Nulliparous (yes; %; N)	77.9 (310)	74.9 (131)	0.43	78.9 (330)	71.6 (111)	0.06
Anovulatory (yes; %; N)	44.8 (178)	51.4 (90)	0.15	54.1 (188)	51.6 (80)	0.16
PCOS (yes; %; N)	32.7 (130)	40.6 (71)	0.07	33.1 (138)	40.6 (63)	0.09
Duration infertility (months; mean; SD)	30.5 (27.7)	25.3 (21.0)	0.01	29.7 (27.1)	26.7 (22.6)	0.22
Vegetable intake (g/day; median; IQR)	116.1 (84.8; 171.4)	114.3 (80.4; 157.1)	0.52	114.3 (78.6; 171.4)	128.6 (92.9; 171.4)	0.15
Fruit intake (g/day median; IQR)	85.7 (42.9; 142.9)	100.0 (57.1; 142.9)	0.50	85.7 (42.9; 142.9)	100.0 (57.1; 142.9)	0.38
Sugary drinks (glasses/day median; IQR)	0.97 (0.23; 2.48)	0.86 (0.22; 2.00)	0.29	0.91 (0.23; 2.39)	0.86 (0.22; 2.08)	0.55
Savoury snacks (handful/week median; IQR)	4.4 (1.8; 9.0)	4.4 (1.8; 12.5)	0.56	4.4 (1.8; 12.5)	4.4 (1.8; 9.0)	0.34
Sweet snacks (portion/week median; IQR)	4.5 (0.9; 6.5)	2.5 (0.9; 6.5)	0.80	4.5 (0.9; 6.5)	2.5 (0.9; 6.5)	0.64
Total MVPA (min/week median; IQR)	372.5 (143.8; 985.0)	380.0 (145.0; 960.0)	0.75	360.0 (140.0; 1010.0)	397.0 (150.0; 903.8)	0.90
Meeting Dutch PA guidelines (yes; %; N)	74.9 (253)	75.2 (121)	0.94	74.7 (268)	75.7 (106)	0.81
Successful weight loss intervention group†						
Yes (%; N)	40.6 (76)	58.1 (43)	0.01	41.4 (79)	57.1 (40)	0.02
No (%; N)	59.4 (111)	41.9 (31)		58.6 (112)	42.9 (30)	
Time between completion intervention period and follow-up approach (years; median; IQR) #	4.40 (3.70; 5.02)	4.49 (3.90; 5.12)	0.24	4.39 (3.70; 5.00)	4.53 (3.95; 5.16)	0.047

* To compare means between both groups, an independent sample t-test was used; to compare medians a Mann Whitney-U test was used; to compare categorical variables a chi-squared test was used. FFQ = food frequency questionnaire; SD = standard deviation; PCOS = Polycystic Ovary Syndrome; g/day = grams per day; MVPA = moderate to vigorous physical activity; min/week =

minutes per week; PA = physical activity.
** BMI was measured by research nurses during hospital visit.
† Successful weight loss was defined as ≥5% weight loss or a BMI≤29kg/m^2 within six months after randomisation into the intervention arm of the LIFEstyle study (N=261 due to missing data regarding weight change).
# Time between completion intervention period and follow up approach was calculated as: date women were approached for follow-up – (randomisation date + 6 months (duration intervention period)) in both the intervention group as well as the control group.
