# Supplementary figures and images for: Preconception lifestyle intervention reduces long term energy intake in women with obesity and infertility: a randomised controlled trial
Source: Int J Behav Nutr Phys Act. 2019 Jan 8;16:3. doi: 10.1186/s12966-018-0761-6 (PMC6325811; doi:10.1186/s12966-018-0761-6)

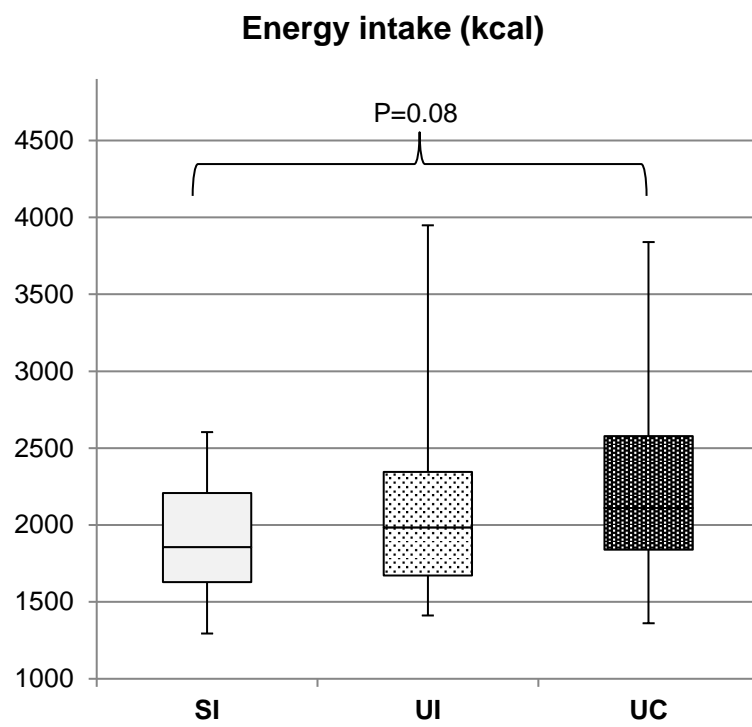

Supplement: Supplementary file 2 — Figure S1. Differences in energy intake (kcal) at follow-up without underreporters and without women who successfully lost weight in the control group (N = 3) or had missing data on weight loss in the control group (N = 16). Differences among women who successfully lost weight during the intervention (SI; N = 29), who were unsuccessful in losing weight during the intervention (UI; N = 24) and women who were unsuccessful in losing weight in the control group (UC; N = 54) were analysed using ANCOVA, corrected for: Caucasian origin (yes/no), education level (categorical: no education or primary school; secondary education; intermediate vocational education; higher vocational education and university), smoking (yes/no) and duration of infertility (months). Tukey post-hoc tests were used to analyse differences within groups. Mean kcal and SD: SI = 1917 kcal (358); UI = 2097 kcal (544); UC = 2234 kcal (583). (PDF 7 kb) [file 12966_2018_761_MOESM2_ESM.pdf]

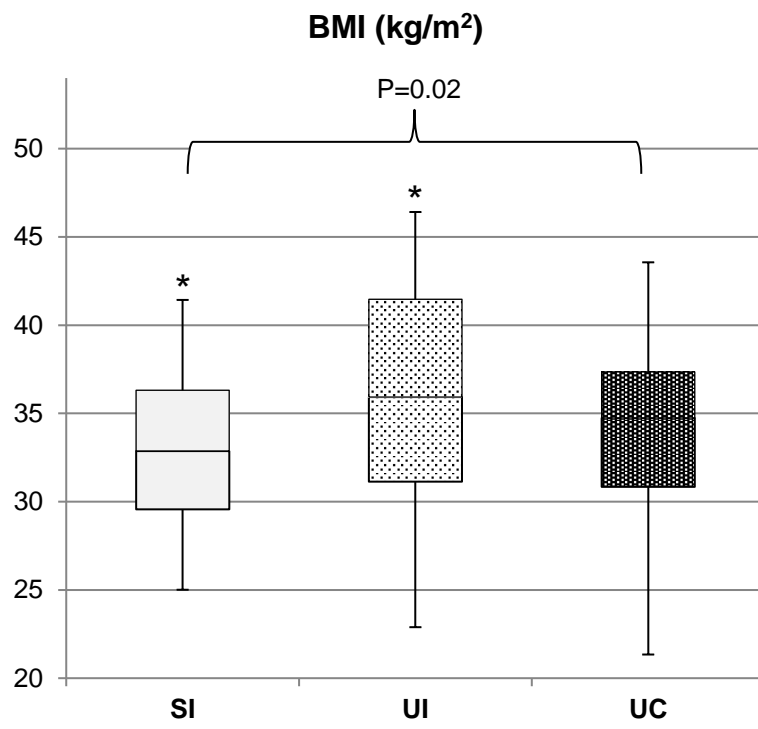

Supplement: Supplementary file 3 — Figure S2. Differences in self-reported BMI (kg/m2) at follow-up without women who successfully lost weight in the control group (N = 3) or had missing data on weight loss in the control group (N = 22). Differences among women who successfully lost weight during the intervention (SI; N = 45), who were unsuccessful in losing weight during the intervention (UI; N = 33) and women who were unsuccessful in losing weight in the control group (UC; N = 70) were analysed using ANCOVA, corrected for: Caucasian origin (yes/no), education level (categorical: no education or primary school; secondary education; intermediate vocational education; higher vocational education and university), smoking (yes/no) and duration of infertility (months). Tukey post-hoc tests were used to analyse differences within groups. Mean BMI and SD: SI = 32.9 kg/m2 (4.0); UI = 36.2 kg/m2 (6.0); UC = 34.0 kg/m2 (4.8). * P-value = 0.01 (PDF 7 kb) [file 12966_2018_761_MOESM3_ESM.pdf]
